# Obsessive compulsive disorder in coroners’ reports

**DOI:** 10.1192/bjo.2021.789

**Published:** 2021-06-18

**Authors:** Himanshu Tyagi, Lisa Quigley

**Affiliations:** 1 UCL Division of Psychiatry, UCL Division of Neurology, UCLH; 2 MSc Student, UCL Division of Psychiatry

## Abstract

**Aims:**

The frequency and burden of suicidality in obsessive-compulsive and related disorders have historically been under-reported, despite research pointing to a significant association between OCD and suicidality. Likewise, OCD is frequently undiagnosed or misdiagnosed. This study looks at coroners’ reports relating to suicides in UK, Australia and Canada in order to:

Explore characteristics of suspected or confirmed cases of OCD in coroners’ reports

Identify instances of possible undiagnosed or misdiagnosed OCD

Identify recurring themes in the reports

**Method:**

1869 publicly available coroners’ reports were accessed from England (n = 200), Scotland (n = 128), Canada (n = 680) and Australia (n = 861). Reports were screened in order to identify individuals who had either a diagnosis of OCD (n = 16), a diagnosis of a related condition (n = 4), or indications of possible undiagnosed OCD (n = 12). Wherever possible, demographic and psychiatric characteristics were extracted for statistical analysis. Qualitative thematic analysis was carried out on selected reports.

**Result:**

32 cases of interest were identified from analysis of coroners’ reports of suicides that took place between the years of 2000 and 2020. Breakdown by country was as follows:

United Kingdom: n = 6 (1.8% of total reports analysed from United Kingdom)

Canada n = 3 (0.4% of total reports analysed from Canada)

Australia n = 23 (2.7% of total reports analysed from Australia)

Among those with possible undiagnosed OCD, common experiences were fear of causing harm, intrusive thoughts of guilt and shame, and compulsive checking and/or reassurance seeking. Further themes included: misdiagnosis, failings in mental health care, stigma and discrimination.

**Conclusion:**

Individuals with OCD are thought to be up to ten times more likely to die by suicide, with this risk increasing in the presence of psychiatric comorbidities. However, OCD remains underdiagnosed, and this may be reflected in the relatively low number of suicides identified for this study where OCD was diagnosed before death. The low numbers may also point to a tendency among both coroners and healthcare professionals to underestimate the association between OCD and suicidality.

Qualitative analysis of the coroners’ reports identified a theme of intolerable distress. This distress was documented most extensively in reports where OCD was strongly indicated but never diagnosed, highlighting the impact of missed, late or incorrect diagnosis.

Notably, nearly all of the reports reveal repeated attempts by the individual to seek help. Despite this, many experienced stigma, mental health service failings and missed opportunities for help in the months preceding their deaths.

